# Supplementary material for: Genomic patterns of progression in smoldering multiple myeloma
Source: Nat Commun. 2018 Aug 22;9:3363. doi: 10.1038/s41467-018-05058-y (PMC6105687; doi:10.1038/s41467-018-05058-y)
Supplement: Supplementary file 1 — Supplementary Information [file 41467_2018_5058_MOESM1_ESM.pdf]

Supplementary Figures

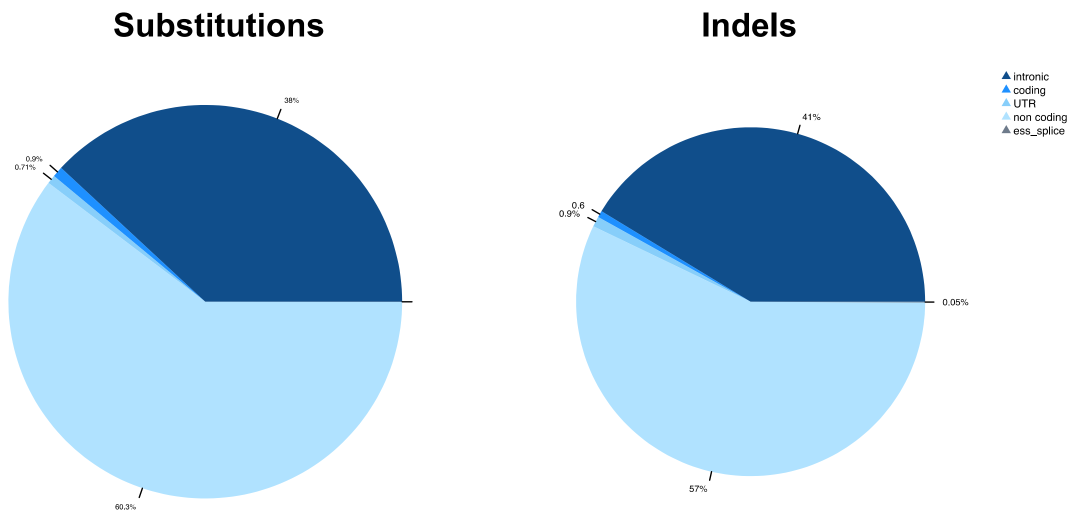

Supplementary Figure 1: Pie chart of variants in the study. Pie charts representing the distribution of all substitutions and indels in the study, broken down by mutation effect.

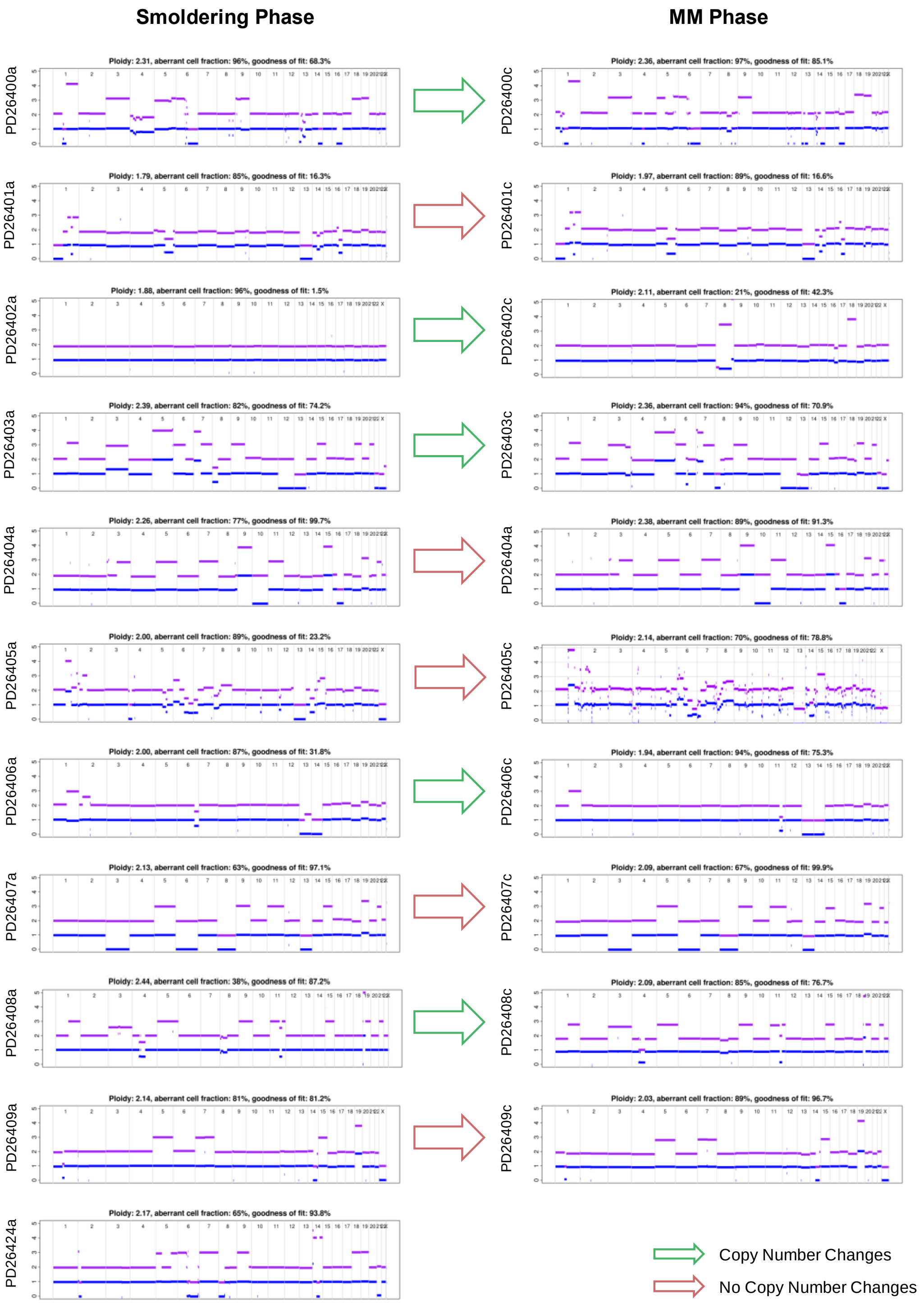

Supplementary Figure 2: Copy number abnormalities. Chromosomal copy-number plots for the paired samples in the study; in each plot, purple = total copy number, blue = copy number of the minor allele. The arrow was colored based on the type of evolution of the major copy number changes (see fig. 2D).

Supplementary Figure 3: Circos plots. Circos plots from all samples in the study. From the external ring to the internal:

- mutations, where the color of each dot represents the mutation class, and the vertical position is function of the intermutational distance;
- indels, where dark green and brown lines represent insertion and deletion respectively;
- copy number variants, where red = deletions, green = gain;
- rearrangements, where blue = inversion, red = deletions, green = ITD, purple = translocation. Only rearrangements with a cancer cell fraction > 10% were annotated.

On the right, from top to bottom: 96-class histogram of distribution of mutations by nucleotide change, in pyrimidine context; barplot of absolute number of mutations; barplot of absolute numbers of rearrangements.

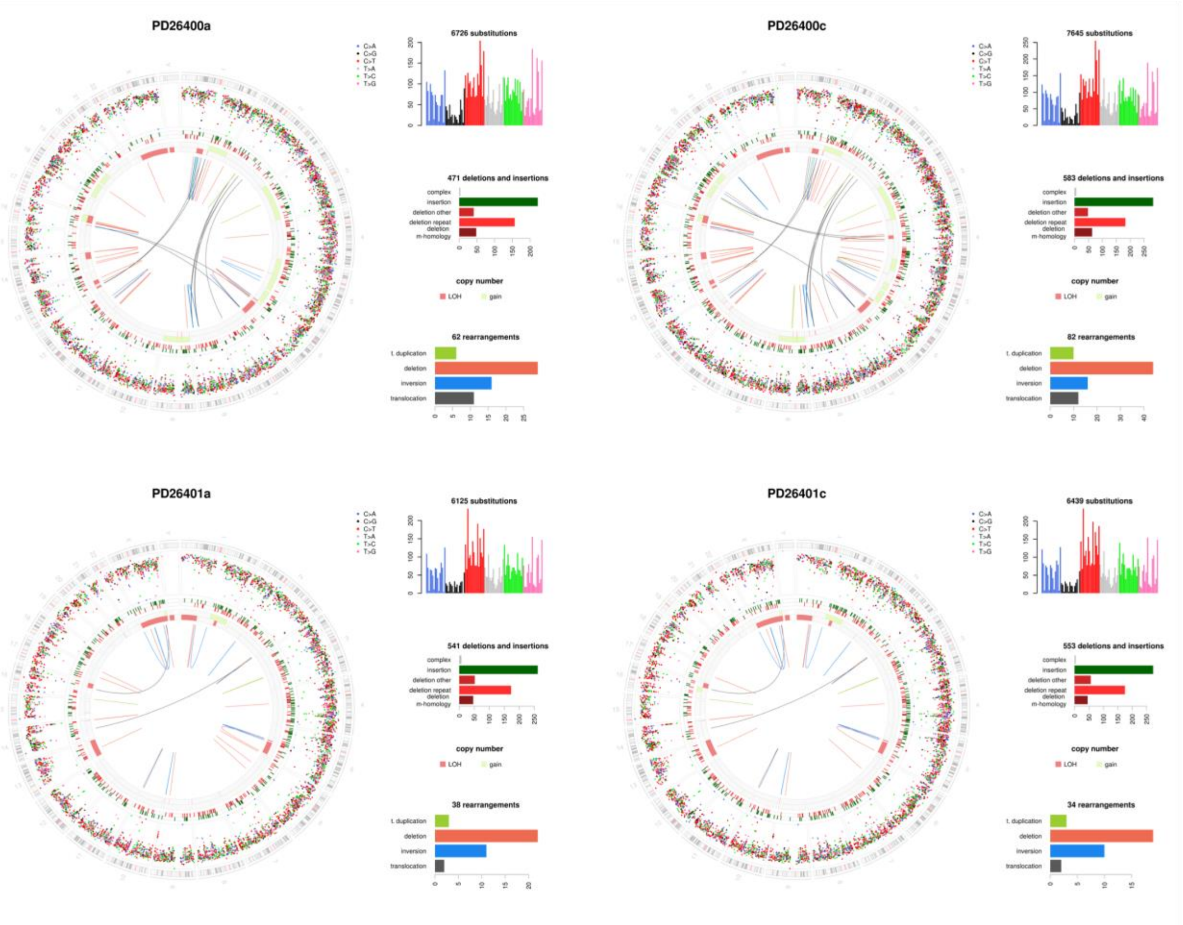

PD26402a

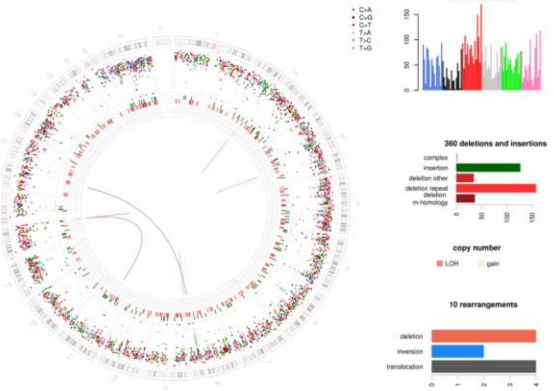

PD26402c

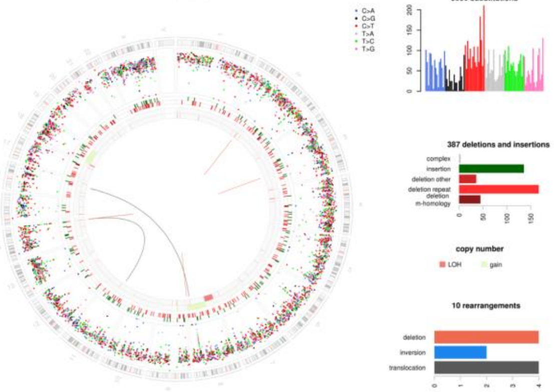

PD26403a

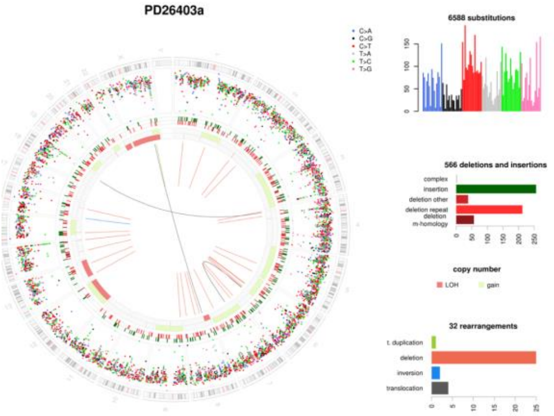

PD26403c

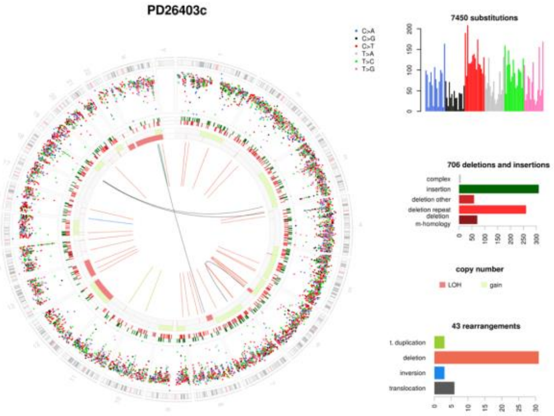

PD26404a

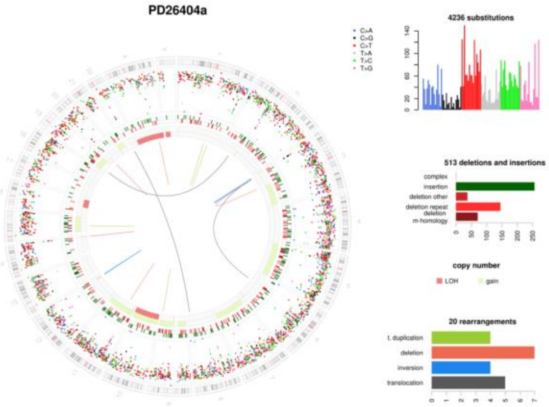

PD26404c

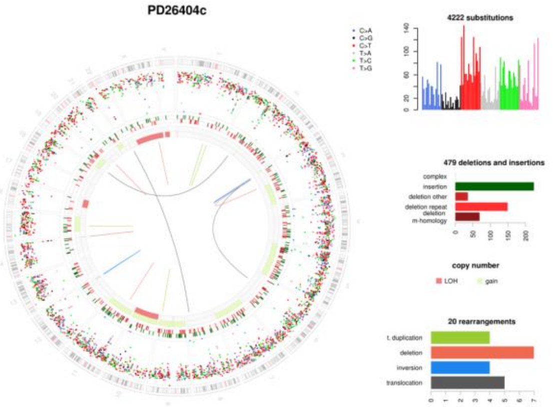

PD26405a

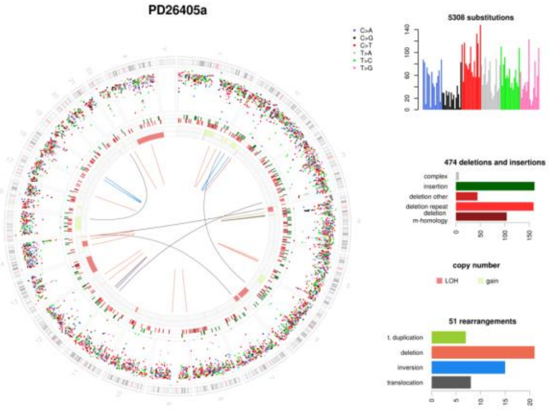

WGA_PD26405c

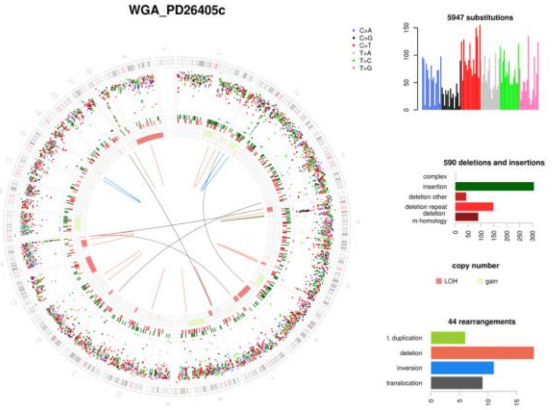

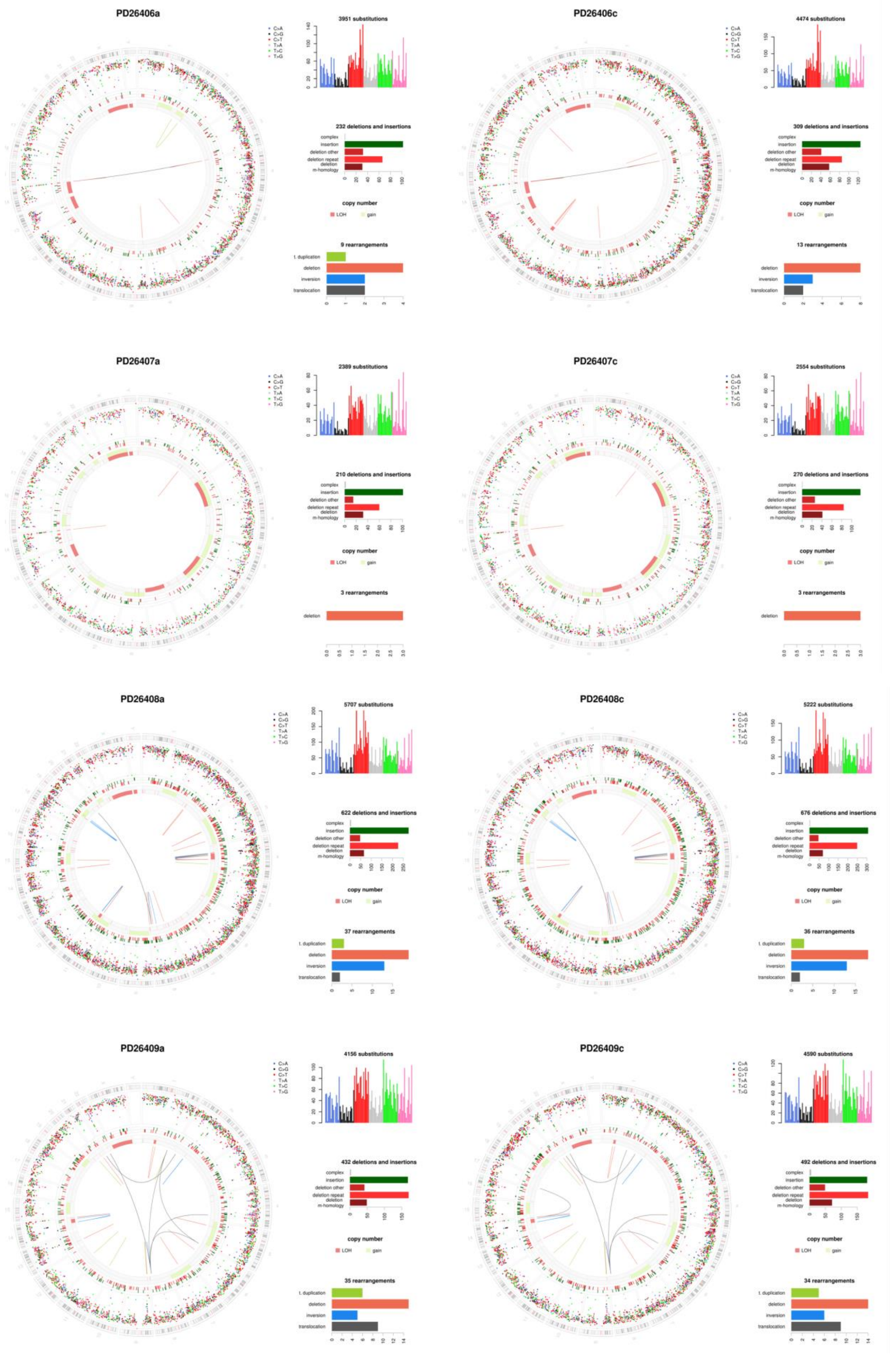

PD26406a
3951 substitutions
232 deletions and insertions
copy number
LOH
gain
9 rearrangements
PD26406c
4474 substitutions
309 deletions and insertions
13 rearrangements
PD26407a
2389 substitutions
210 deletions and insertions
3 rearrangements
PD26407c
2554 substitutions
270 deletions and insertions
3 rearrangements
PD26408a
5707 substitutions
622 deletions and insertions
37 rearrangements
PD26408c
5222 substitutions
676 deletions and insertions
36 rearrangements
PD26409a
4156 substitutions
432 deletions and insertions
35 rearrangements
PD26409c
4590 substitutions
492 deletions and insertions
34 rearrangements
C>A
C>G
C>T
T>A
T>C
T>G
complex
insertion
deletion other
deletion repeat
deletion m-homology
t. duplication
deletion
inversion
translocation

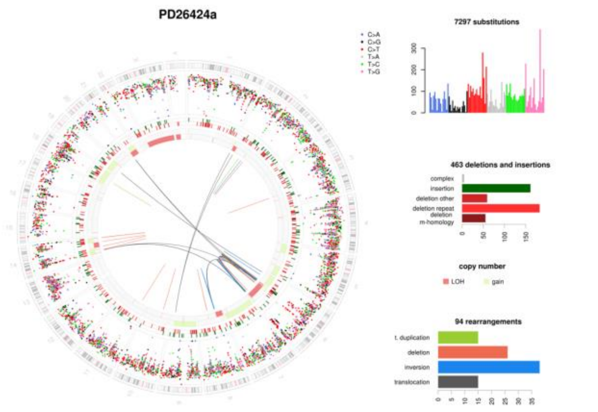
PD26424a
C>A
C>G
C>T
T>A
T>C
T>G
7297 substitutions
0
100
200
300
463 deletions and insertions
complex
insertion
deletion other
deletion repeat
deletion
m-homology
0
50
100
150
copy number
LOH
gain
94 rearrangements
t. duplication
deletion
inversion
translocation
0
5
10
15
20
25
30
35

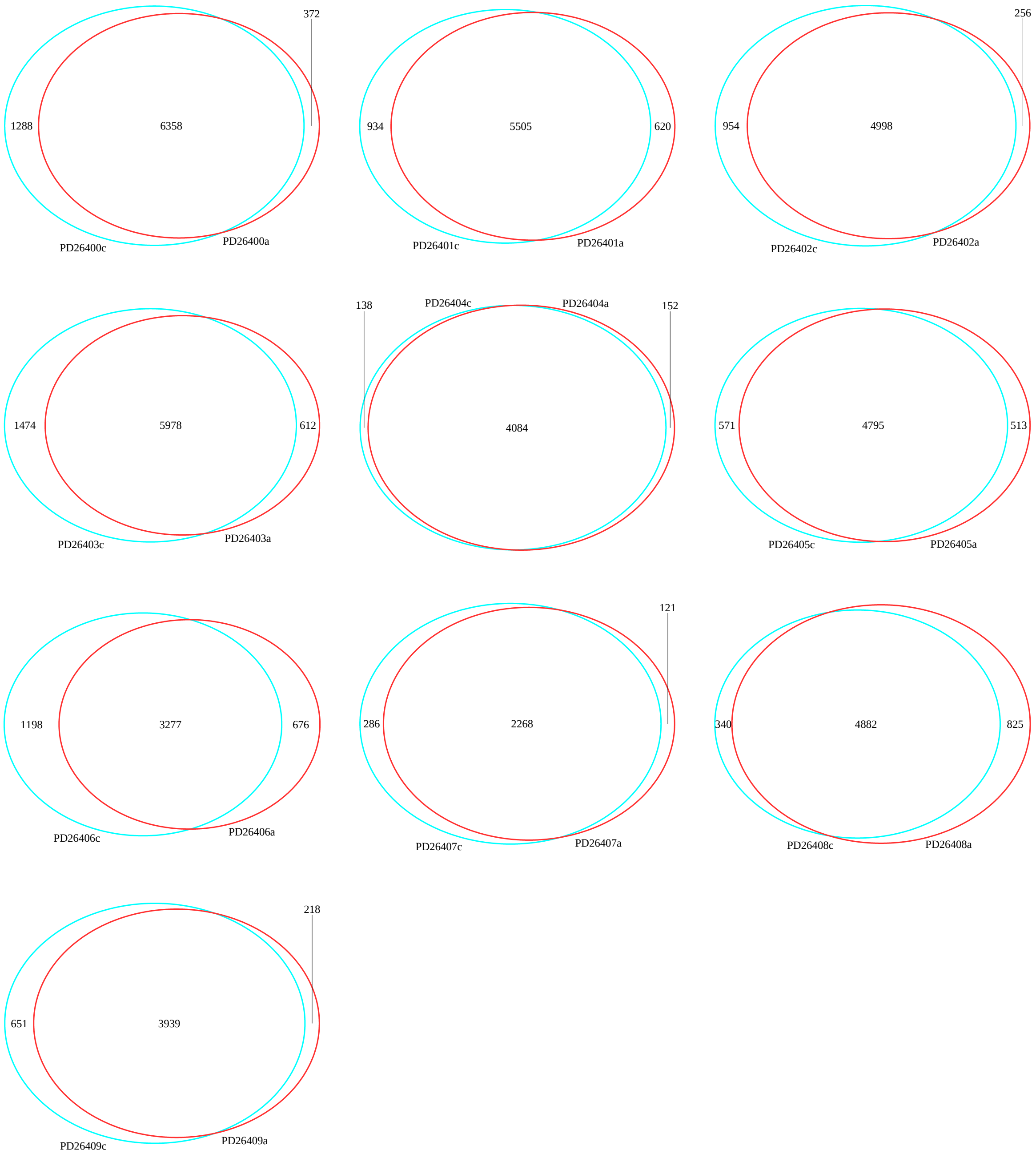

Supplementary Figure 4: Overlap of mutations in paired samples. Venn plots of mutations in each sample pair. Red, SMM sample; blue, MM sample.

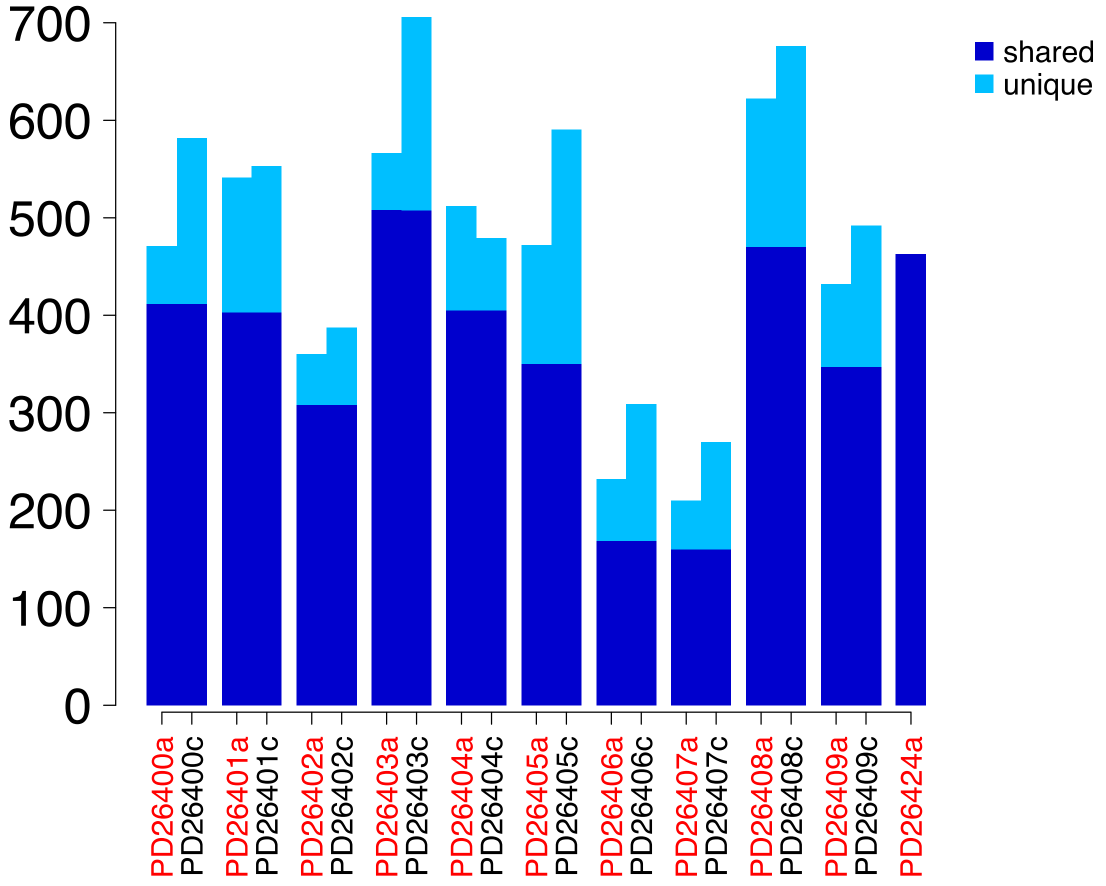

Supplementary Figure 5: Shared and unique indels in paired samples

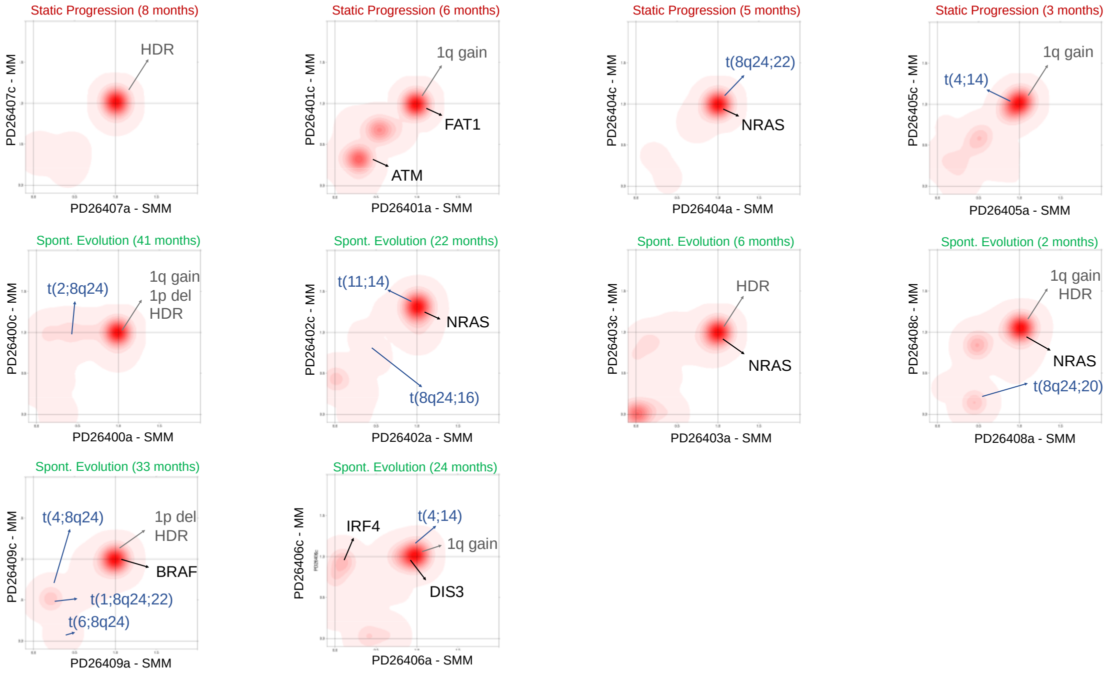

Supplementary Figure 6: Dirichlet clustering of mutations in the study. Dirichlet plots of all pairs in the study, marked with the location of the main driver events.

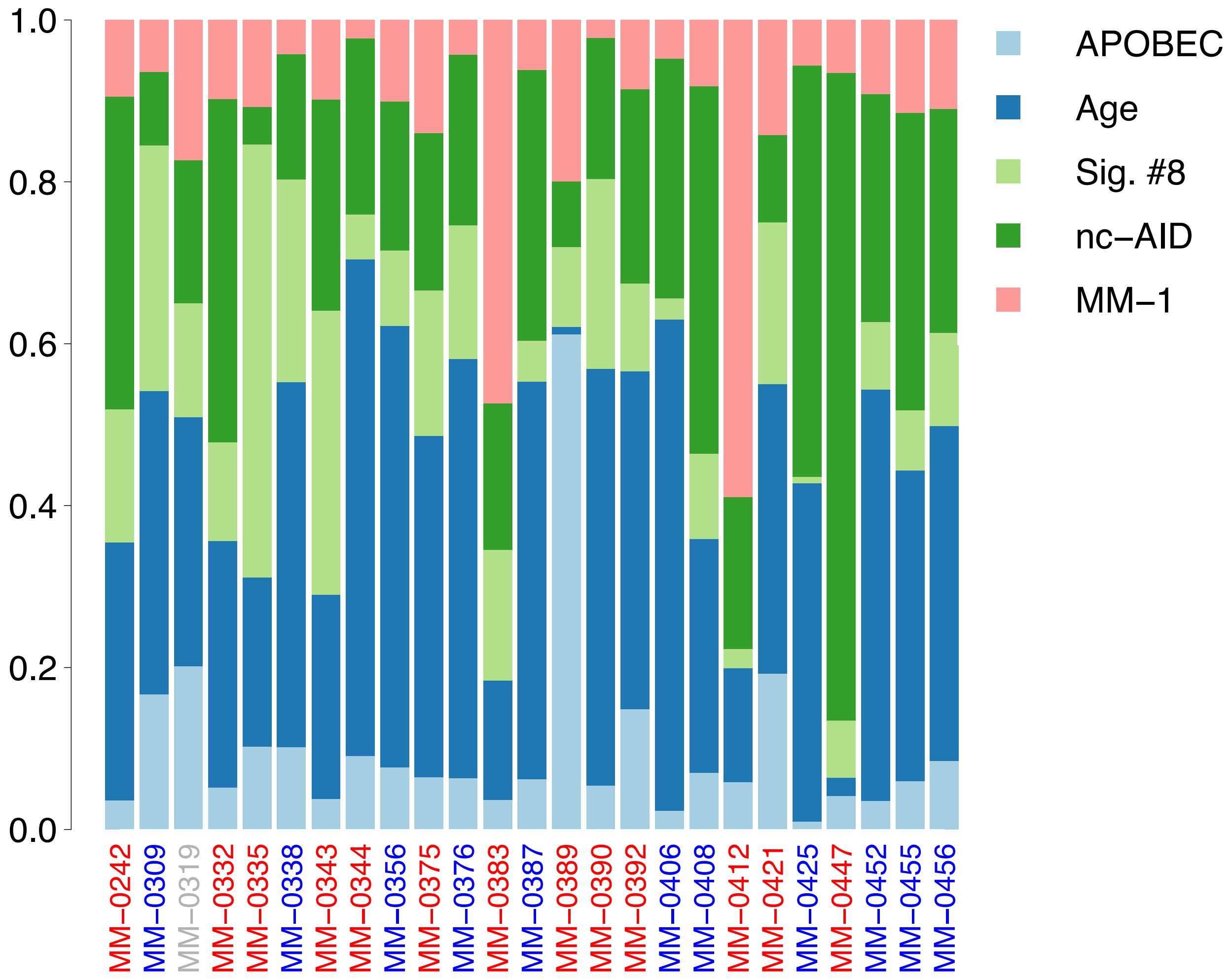

Supplementary Figure 7: Validation of mutational processes in an independent cohort. Barplot representing the contribution of each mutational signature in the validation cohort of public MM WGS data (DbGaP accession n. phs000348.v2.p1). Unique patient identifiers in red represent MM cases at relapse; blue, MM cases at diagnosis; grey, no information available.

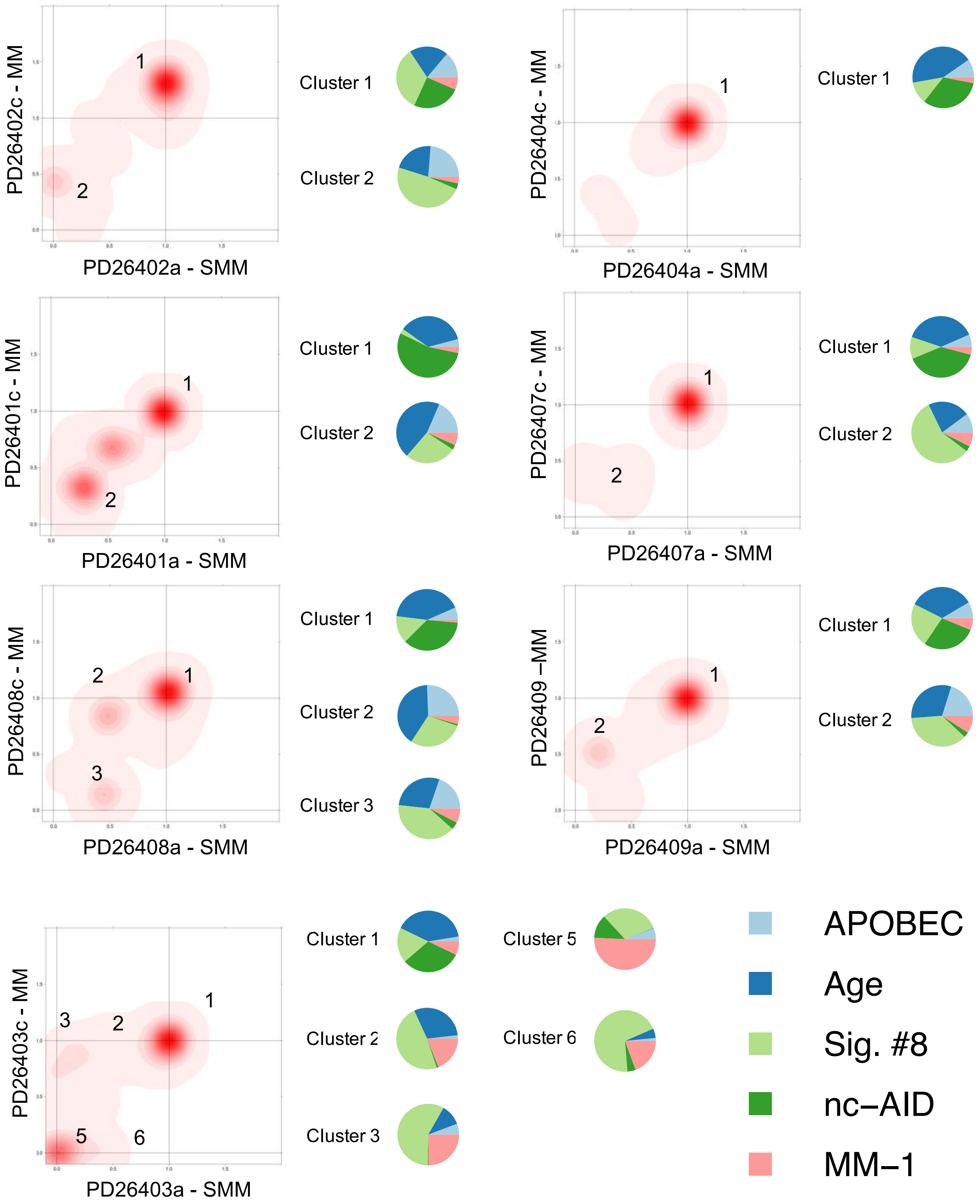

Supplementary Figure 8: Relative contribution of mutations over time**.** Mutational signature contribution during progression in the 7 patients not included in figure 6**.**

Supplementary Tables

Supplementary Table 1 clinical profiles pf patients at SMM diagnosis and symptomatic evolution.

Sample	RIsk	Date of Birth	Date of Sampling	Age	Sex	MC SMM*	Isotype	sFLC Ratio	BMPC SMM (%)	Imaging[&]	MC MM*	MM criteria at{Rajkumar:2011dn} MM diagnosis
**PD26400a**	High	26/07/1939	19/08/2008	69	M	14	IgG K	NA	33	Neg	40	Anemia
**PD26401a**	High	02/07/1936	30/03/2010	74	M	36	IgG K	NA	21	Neg	37	Anemia
**PD26402a**	High	05/07/1955	20/04/2010	55	F	41	IgG K	1.11	40	Neg	76	Bone, Anemia
**PD26403a**	Standard	20/05/1953	21/04/2010	57	F	22	IgG K	79	29	Neg	62	Bone
**PD26404a**	High	23/07/1946	09/09/2010	64	M	42	IgG K	NA	37	Neg	42	Bone
**PD26405a**	High	16/08/1946	03/11/2010	64	F	40	IgA K	Normal	25	Neg	46	Anemia, bone
**PD26406a**	High	08/01/1949	18/01/2010	61	M	31	IgG K	NA	NA	Neg	82	Hypercalcemia, renal failure
**PD26407a**	Standard	28/06/1962	23/02/2011	49	M	19	IgG K	Normal	39	Neg	29	Bone
**PD26408a**	High	22/03/1959	26/10/2011	53	M	31	IgG K	Normal	31	Neg	37	Bone
**PD26409a**	Standard	09/09/1948	21/03/2012	64	F	25	IgG K	NA	10	Neg	32	Anemia, Malignant Osteoporosis
**PD26424a**	Ultra-high	26/08/1947	21/10/2010	63	M	46	IgG K	768.18	61	Neg	NA	Osteopenia at SMM stage

*g/dL

[&]X-Ray scan

BMPC = bone marrow plasma cells.

MC = monoclonal component

sFLC = serum free light chain

Supplementary Table 2 Samples characteristics and median whole genome sequencing coverage.

Sample	Median Coverage	Disease Phase[&]	Date of Birth	Date Sampling	Tissue*	Age	Sex	ACF[%]	Ploidy
PD26400a	38.7	SMM	26/07/1939	19/08/2008	BM	69	M	0.96	2.31
PD26400b	36.9	Normal Match			PB		-	-	-
PD26400c	38.5	MM	26/07/1939	12/01/2012	BM	72	M	0.97	2.36
PD26401a	45.5	SMM	02/07/1936	30/03/2010	BM	74	M	0.85	1.79
PD26401b	36.5	Normal Match			PB		-	-	-
PD26401c	43.1	MM	02/07/1936	06/10/2010	BM	74	M	0.89	1.97
PD26402a	43.4	SMM	05/07/1955	20/04/2010	BM	55	F	0.96	1.88
PD26402b	33.7	Normal Match			PB		-	-	-
PD26402c	41.0	MM	05/07/1955	20/02/2012	BM	57	F	0.21	2.11
PD26403a	39.3	SMM	20/05/1953	21/04/2010	BM	57	F	0.82	2.39
PD26403b	34.4	Normal Match			PB		-	-	-
PD26403c	40.0	MM	20/05/1953	19/10/2010	BM	57	F	0.94	2.36
PD26404a	41.1	SMM	23/07/1946	09/09/2010	BM	64	M	0.77	2.26
PD26404b	36.1	Normal Match			PB		-	-	-
PD26404c	36.1	MM	23/07/1946	23/02/2011	BM	65	M	0.89	2.38
PD26405a	42.6	SMM	16/08/1946	03/11/2010	BM	64	F	0.89	2.0
PD26405b	35.5	Normal Match			PB		-	-	-
WGA_PD26405c	36.8	MM	16/08/1946	18/01/2011	BM	64	F	0.88	2.05
PD26406a	35.2	SMM	08/01/1949	18/01/2010	BM	61	M	0.87	2.0
PD26406b	32.1	Normal Match			PB		-	-	-
PD26406c	39.3	MM	08/01/1949	17/01/2012	BM	63	M	0.94	1.94
PD26407a	36.0	SMM	28/06/1962	23/02/2011	BM	49	M	0.63	2.13
PD26407b	34.3	Normal Match			PB		-	-	-
PD26407c	43.8	MM	28/06/1962	12/10/2011	BM	49	M	0.67	2.09
PD26408a	47.8	SMM	22/03/1959	26/10/2011	BM	53	M	0.38	2.44
PD26408b	29.1	Normal Match			PB		-	-	-
PD26408c	46.7	MM	22/03/1959	26/12/2011	BM	53	M	0.85	2.09
PD26409a	41.4	SMM	09/09/1948	21/03/2012	BM	64	F	0.81	2.14
PD26409b	34.5	Normal Match			PB		-	-	-
PD26409c	45.1	MM	09/09/1948	28/01/2014	BM	65	F	0.89	2.03
PD26424a	37.3	SMM	26/08/1947	21/10/2010	BM	63	M	0.65	2.17
PD26424b	36.1	Normal Match			PB			-	-

[&] SMM= Smoldering Multiple Myeloma; MM = Multiple Myeloma; MGUS = monoclonal gammopathy of undetermined significance
*BM = bone marrow; PB = peripheral blood
[%]ACF = aberrant cell fraction

Supplementary Table 3 Dirichlet clusters composition, size and signature contribution for each sample

Patients	Cluster number	% SMM cells	% MM cells	estimated no of mutations	n. of mutations assigned	APOBEC	Age	Sis. 8	nc-AID	MM1
PD26400	1	0.99	0.99	5230	5507	0.10	0.22	0.30	0.35	0.01
PD26400	2	0.56	0.99	917	885	0.31	0.19	0.36	0.05	0.07
PD26400	3	0.19	0.99	662	666	0.34	0.22	0.36	0.01	0.05
PD26400	4	0.09	0.3	475	468	0.32	0.12	0.48	0.03	0.03
PD26401	1	0.99	0.99	2873	2957	0.04	0.36	0.02	0.54	0.03
PD26401	2	0.38	0.46	3660	3770	0.18	0.45	0.27	0.02	0.06
PD26401	3	0.74	0.72	197	3	-	-	-	-	-
PD26402	1	1	1.28	5027	5030	0.13	0.20	0.33	0.25	0.06
PD26402	2	0.09	0.42	1017	1022	0.23	0.21	0.48	0.03	0.03
PD26403	1	0.98	0.99	5686	5853	0.02	0.40	0.18	0.31	0.07
PD26403	2	0.54	0.94	437	269	0.01	0.30	0.48	0.01	0.18
PD26403	3	0.13	0.78	1180	1178	0.01	0.04	0.69	0.04	0.19
PD26403	4	0.44	0.47	28	29					
PD26403	5	0.05	0.01	3449	3450	0.05	0.002	0.30	0.12	0.50
PD26403	6	0.42	0.01	283	283	0.05	0.11	0.58	0.004	0.24
PD26404	1	0.99	0.99	4135	4135	0.09	0.43	0.11	0.32	0.03
PD26404	2	0.31	0.24	48	48					
PD26405	1	0.98	1	3984	4100	0.09	0.32	0.24	0.26	0.06
PD26405	2	0.6	0.64	440	214	0.10	0.02	0.73	0.11	0.02
PD26405	3	0.35	0.4	1381	1490	0.19	0.003	0.71	0.02	0.06
PD26406	1	0.95	1.01	3177	3209	0.14	0.231	0.17	0.33	0.11
PD26406	2	0.08	0.87	1008	1011	0.26	0.04	0.37	0.22	0.08
PD26406	3	0.49	0.04	562	562	0.42	0.18	0.08	0.14	0.15
PD26406	4	0.08	0.39	123	101	0.37	0.08	0.39	0.04	0.10
PD26407	1	1.01	1.01	2291	2300	0.06	0.38	0.11	0.39	0.04
PD26407	2	0.27	0.35	242	236	0.10	0.22	0.57	0.02	0.07
PD26408	1	1.02	1.05	3608	3757	0.06	0.41	0.14	0.36	0.01
PD26408	2	0.57	0.86	1392	1250	0.19	0.28	0.40	0.03	0.07
PD26408	3	0.47	0.16	794	787	0.25	0.40	0.28	0.01	0.04
PD26409	1	0.97	0.98	3968	3968	0.08	0.34	0.22	0.28	0.06
PD26409	2	0.24	0.46	817	817	0.20	0.30	0.36	0.027	0.09

Supplementary Table 4 Clinical and sequencing data of the cohort of additional 19-MM WGS included for the signature analysis

Sample_ID	Coverage	Age	Gender	Disease Phase*	Cytogenetic
**PD26410d**	37.70	66	M	MM RR	HRD
**PD26411a**	39.26	65	M	MM RR	HRD
**PD26412a**	34.39	50	M	MM RR	HRD
**PD26414a**	35.18	54	F	MM RR	t(4;14)
**PD26415c**	38.41	55	M	MM RR	HRD
**PD26416d**	36.12	52	F	MM RR	HRD
**PD26418a**	40.35	71	M	MM RR	t(11;14)
**PD26419a**	36.31	65	F	MM DG	na
**PD26420a**	40.69	56	M	MM RR	t(11;14)
**PD26422d**	37.67	70	M	MM RR	t(11;14)
**PD26423e**	33.93	54	M	MM RR	HRD
**PD26425e**	36.54	63	M	MM RR	t(11;14)
**PD26426e**	33.50	77	M	MM RR	HRD
**PD26427a**	34.52	51	F	MM DG	t(11;14)
**PD26428a**	29.63	41	M	MM DG	t(11;14)
**PD26429a**	32.90	64	F	MM DG	HRD
**PD26432c**	34.35	61	M	MM RR	HRD
**PD26434c**	34.46	58	M	MM DG	t(11;14)
**PD26435c**	38.05	65	M	MM RR	HRD

*MMR = Relapse/Refractory MM; MM DG = newly diagnosed MM
